# Beyond targeting amplified MDM2 and CDK4 in well differentiated and dedifferentiated liposarcomas: From promise and clinical applications towards identification of progression drivers

**DOI:** 10.3389/fonc.2022.965261

**Published:** 2022-09-02

**Authors:** Giuliana Cassinelli, Sandro Pasquali, Cinzia Lanzi

**Affiliations:** ^1^ Molecular Pharmacology Unit, Department of Applied Research and Technological Development, Fondazione Istituto di Ricovero e Cura a Carattere Scientifico (IRCCS) Istituto Nazionale dei Tumori, Milan, Italy; ^2^ Sarcoma Service, Department of Surgery, Fondazione Istituto di Ricovero e Cura a Carattere Scientifico (IRCCS) Istituto Nazionale dei Tumori, Milan, Italy

**Keywords:** well differentiated liposarcoma, dedifferentiated liposarcoma, soft tissue sarcoma, MDM2, CDK4, XPO1, receptor tyrosine kinase, investigational therapy

## Abstract

Well differentiated and dedifferentiated liposarcomas (WDLPS and DDLPS) are tumors of the adipose tissue poorly responsive to conventional cytotoxic chemotherapy which currently remains the standard-of-care. The dismal prognosis of the DDLPS subtype indicates an urgent need to identify new therapeutic targets to improve the patient outcome. The amplification of the two driver genes *MDM2* and *CDK4*, shared by WDLPD and DDLPS, has provided the rationale to explore targeting the encoded ubiquitin-protein ligase and cell cycle regulating kinase as a therapeutic approach. Investigation of the genomic landscape of WD/DDLPS and preclinical studies have revealed additional potential targets such as receptor tyrosine kinases, the cell cycle kinase Aurora A, and the nuclear exporter XPO1. While the therapeutic significance of these targets is being investigated in clinical trials, insights into the molecular characteristics associated with dedifferentiation and progression from WDLPS to DDLPS highlighted additional genetic alterations including fusion transcripts generated by chromosomal rearrangements potentially providing new druggable targets (e.g. NTRK, MAP2K6). Recent years have witnessed the increasing use of patient-derived cell and tumor xenograft models which offer valuable tools to accelerate drug repurposing and combination studies. Implementation of integrated “multi-omics” investigations applied to models recapitulating WD/DDLPS genetics, histologic differentiation and biology, will hopefully lead to a better understanding of molecular alterations driving liposarcomagenesis and DDLPS progression, as well as to the identification of new therapies tailored on tumor histology and molecular profile.

## Introduction

Well differentiated (WD) and dedifferentiated (DD) liposarcomas (LPS) represent the most frequent adipose tissue tumors occurring preferably in adults, particularly in the retroperitoneum and extremities (1). WDLPS and DDLPS exhibit different aggressive potential reflecting their morphologic diversity. WDLPS, a low-grade tumor characterized by malignant adipocytes, may recur locally after surgery, a condition potentially lethal when presenting in the retroperitoneum. DDLPS is a high-grade non-lipogenic malignancy with propensity to both recur locally and metastasize, particularly when located in the retroperitoneum compared to extremity and trunk wall (2–4). WD and DD components often coexist, suggesting that WDLPS and DDLPS represent evolution states of one disease. Indeed, they share peculiar supernumerary ring and giant marker chromosomes including amplified sequences of 12q13-15 (5), which cause overexpression of genes that act as oncogenic drivers (i.e. *MDM2* and *CDK4*) and represent adipocytic differentiation/diagnostic markers (i.e. *HMGA2* and *CPM*) (6–8). In comparison to WDLPS, DDLPS generally harbor additional epigenetic and genetic alterations (6–8). DDLPS may undergo heterologous differentiation associated with a more aggressive phenotype mainly evident when rhabdomyoblastic traits are acquired (9–12).

Surgery represents the curative treatment for localized WDLPS/DDLPS. Neoadjuvant radiation therapy has been suggested to reduce the risk of local recurrence (13) occurring in about one in three-four patients (2, 3). LPS-specific therapeutic options for patients who develop local recurrence and distant metastasis are lacking and both WDLPS and DDLPS are poorly responsive to either conventional cytotoxic chemotherapy or clinically tested targeted therapies (14, 15). These issues underline the need to identify new actionable targets and biology-driven LPS-specific therapeutic approaches to impact patient outcomes (8, 16, 17).

Herein, after briefly summarizing the current standard-of-care and recent findings from clinical investigations on new therapeutic strategies for WD/DDLPS, we focus on the rationale behind emerging treatment options exploiting potential vulnerabilities based on LPS biology. We do not address here immunotherapeutic approaches, which are summarized in other reviews (8, 18).

## Conventional systemic and histotype-specific therapies

Therapies used in most soft tissue sarcomas (STS) derive from studies that investigated a general STS patient population. Mirroring STS, anthracycline-based therapies remain the first-line treatment for advanced DDLPS (19). However, Phase II/III trials showed that the low tumor response to doxorubicin (<10%) was enhanced when combined with high-dose ifosfamide (22%) (15). High-dose ifosfamide has shown effectiveness in recurrent WD/DDLPS even after previous treatment with anthracyclines plus ifosfamide. Gemcitabine or docetaxel monotherapies are also commonly used second-line treatments (14, 20, 21). The addition of docetaxel to gemcitabine improved treatment efficacy compared to gemcitabine alone but, due to the increased toxicity, this option needs a careful patient selection (22). An increasing knowledge about drug sensitivities of STS histologies and results from several retrospective and prospective clinical trials have prompted histology-driven treatments (19, 23). Two marine-derived cytotoxic drugs, trabectedin and eribulin, have been approved by FDA and EMA for the treatment of metastatic WDLPS/DDLPS following phase II studies and further comparative phase III trials showing improved benefit over dacarbazine (24, 25). However, trabectedin impact on progression-free survival did not translate into a survival benefit (26), while eribulin produced only a limited improvement of overall survival without improving progression-free survival (27). Retrospective evidence suggested that low grade DDLPS may benefit from trabectedin while ifosfamide may be more active for high grade DDLPS (28). Nevertheless, clinical, histological and molecular features, as well as predictive biomarkers guiding the selection of patients for these treatments and improving their therapeutic index are lacking (29).

## Overexpressed biomarkers as therapeutic targets: MDM2 AND CDK4

*MDM2* and *CDK4* amplification in over 90% of WDLPS and DDLPS, besides representing a diagnostic tool, provides the rationale for evaluating novel therapies (6, 16, 30, 31).

*MDM2* encodes an E3 ubiquitin-protein ligase that binds p53 promoting its proteasome-mediated degradation thus negatively regulating its tumor suppressor function (32, 33). *MDM2* knockdown heavily impacted DDLPS cell proliferation (34, 35). Likewise, DDLPS cells underwent cell cycle arrest and apoptosis after exposure to nutlins (e.g. nutlin 3a, idasanutlin) (36–38), a prototypical class of MDM2 antagonists specifically designed to block the interaction between MDM2 and p53 (32, 33) (**Table 1**). DDLPS cells harboring wt p53 exhibited a higher responsiveness to MDM2 antagonists belonging to different chemical classes (i.e. nutlin3a, idasanutlin, siremadlin) (**Table 1**) with respect to p53-mutant cell lines (35, 48). A comparative study demonstrated that the higher effectiveness of the MDM2 inhibitor SAR405838 (**Table 1**), compared to its analogue MI-219 and nutlin 3a in inhibiting DDLPS cell growth and inducing apoptosis, relied on the presence of both wt p53 and MDM2 amplification (49). Gene expression analyses implicated restoration of the p53 pathway and reactivation of pro-apoptotic genes in the potent antitumor activity of SAR405838 against a DDLPS xenograft. The MDM2-p53 inhibitor BI-907828 (**Table 1**) induced tumor regression in two DDLPS PDX models with long lasting effect and a complete pathological response in one of them (50).

**Table 1:** Investigational drugs in well differentiated and dedifferentiated liposarcoma.

Drug names (corporation/sponsor)	Primary targets	Clinical development status in cancer	Refs
RG7112, (RO5045337) *(Hoffman-Roche)*	p53-MDM2 binding	Phase I/Ib in advanced solid, hematologic tumors	(32, 33)
idasanutlin (RG7388, (RO5503781)*(Hoffman-Roche)*	p53-MDM2 binding	Phase I/II/III study in advanced solid tumors and AML	(32, 33)
SAR405838 (MI-77301) *(Sanofi-Aventis)*	p53-MDM2 binding	Phase I in advanced solid tumors and MM	(32, 33)
siremadlin (HDM 201) *(Novartis)*	p53-MDM2 binding	Phase I/II in solid and hematological tumors	(33)
BI-907828 *(Boehringer Ingelheim)*	p53-MDM2 binding	A Phase Ia/Ib, in Advanced or metastatic solid tumors	(33)
palbociclib (PD0332991) *(Pfizer)*	CDK4; CDK6	Approved for ER-positive breast cancer	(39–41)
ribociclib (NPV-LEE011) *(Novartis)*	CDK4; CDK6	Approved for ER-positive breast cancer	(39–41)
abemaciclib (LY2835219) *(Eli Lilly)*	CDK4; CDK6, CDK9	Approved ER-positive breast cancer	(39–41)
erdafenib (JNJ-42756493) *(Janssen)*	FGFR1-4	Approved for locally advanced or metastatic urothelial carcinoma	(42, 43)
infigratinib (NPV-BGJ398) *(Novartis)*	FGFR1-3	Approved for unresectable or metastatic cholangiocarcinoma	(43, 44)
LY2874455*(Eli-Lilly)*	FGFR1-4	Phase I advanced-stage solid tumors	(42, 43)
tepotinib (EMD1214063) *(Merck)*	Met	Approved for NSCLC	(45)
crizotinib (PF-02341066) *(Pfizer)*	Met, ALK, ROS	Approved for NSCLC, ALCL	(45)
forentinib (XL880) *(GlaxoSmithKline)*	Met, VEGFR2	Phase I NSCLC	(45)
ponatinib (AP24534) *(Incyte/Takeda)*	BCR-ABL, VEGFR2-3, FGFR1-2, Flt3	Approved CML, ALL	(45)
apatinib (YN968D1) *(Hengrui Medicine)*	VEGFR2, src, c-Kit	Approved for gastric cancer	(45)
pazopanib (GW786034) *(Novartis)*	VEGFR1-3, PDGFR, c-Kit, FGFR1/3	Approved for GIST, pancreatic neuroendocrine tumor, metastatic RCC	(45)
selinexor (KPT-330) *(Karyopharm Therapeutics)*	XPO1	Approved for MM, DLBCL	(46)
AMG900*(Amgen)*	AURKA/B	Phase I in advanced solid tumors and AML	(47)
alisertib (MLN8237) *(Millennium Pharmaceuticals)*	AURKA	Phase I/II in solid and hematological tumors	(47)

ALCL, Anaplastic Large-Cell Lymphoma; ALL, Acute lymphocytic leukemia; AML, Acute Myeloid Leukemia; CML, chronic Myeloid lymphocytic leukemia; DLBCL, diffuse large B cell lymphoma; ER, estrogen receptor; GIST, Gastrointestinal stromal tumor; MM, Multiple Myeloma; NSCLC, Non-Small Cell Lung Cancer; RCC, Renal Cell Carcinoma.

Inhibition of histone deacetylases can affect MDM2 expression/function by acting at both transcriptional and post-translational levels (51, 52). Indeed, in DDLPS models, MDM2 downmodulation induced by histone deacetylase inhibitors such as vorinostat and romidepsin has been associated with antitumor activity *in vitro* and *in vivo* (35, 53). Interestingly, the antitumor activity of the proteasome inhibitor bortezomib against a DDLPS PDX was also associated with MDM2 downmodulation (54).

Preclinical studies suggested rationale-based drug combinations to improve the antitumor efficacy of MDM2-targeting agents. Given the aberrant activation of the PI3K/AKT/mTOR pro-survival pathway observed in DDLPS (55), idasanutlin was tested in combination with the PI3K/mTOR inhibitor NVP-BEZ235 resulting in enhanced cell growth inhibition, apoptotic cell death, and reduction of tumor growth rate (38). Roy et al. (48) described a p53-dependent paradoxical activation of ERK pathway as a mechanism of resistance triggered by idasanutlin. Mechanistically, reactive oxygen species production, due to drug-induced mitochondrial translocation of p53, promotes the activation of receptor tyrosine kinases (RTKs) (e.g. IGF1R, PDGFRβ) and the downstream MEK-1/ERK pathway. The combination of MDM2 antagonists with the MEK1/2 inhibitor GSK112021B synergistically inhibited cell growth and potentiated apoptotic cell death. In mice bearing wt p53 DDLPS xenografts, this drug combination decreased tumor growth and increased survival.

*CDK4* encodes cyclin-dependent kinase 4, which allows G1/S cell cycle progression by phosphorylating Rb. The clinical availability of CDK4/6 inhibitors fostered exploration of CDK4 as therapeutic target in DDLPS (39–41). Both genetic and pharmacological downmodulation of CDK4 inhibited DDLPS cell proliferation inducing cell cycle arrest, apoptosis, and antitumor activity *in vivo* (34, 38, 56, 57). The CDK4 inhibitor ribociclib significantly delayed DDLPS xenograft growth, an effect associated with decreased Rb phosphorylation, BrdU incorporation and ^18^F-Fluorodeoxy-D-glucose uptake (56). CDK4/6 inhibitors can induce a senescent-like cell state which could variably impact tumor growth/progression (58). In WD/DDLPS cell lines, CDK4 inhibitors (i.e. palbociclib, ribociclib, abemaciclib) (**Table 1**) induced senescence associated with accumulation of unphosphorylated Rb and post-translational downregulation of MDM2 (57). MDM2 loss was directly implicated in the senescent program triggered by treatment with CDK4 inhibitors. Notably, a lower expression of MDM2 in tumor biopsies after treatment with palbociclib was associated with tumor response. These findings suggested a favorable interaction between CDK4 inhibitors and agents affecting MDM2 expression/function. Indeed, palbociclib sensitized DDLPS cells to apoptosis induced by idasanutlin through promoting a full activation of the p53 pathway (38). Moreover, this combination reduced DDLPS xenografts growth and improved mice survival. The enhanced antitumor activity observed upon co-treatment with suboptimal doses of ribociclib and siremadlin prompted the clinical evaluation of this combination (59) (**Table 2**).

**Table 2:** Summary of results from clinical trials that tested innovative treatment options for patients with metastatic WDLPS and DDLPS.

DrugsCorporation/sponsor	Study phase	Population	Treatment line	N° of patients	Main findings in LPS	Refs
p53-MDM2 binding inhibitor AMG-232 *(Kartos therapeutics)*	I	Metastatic WDLPS/DDLPS	>3	48 non-adipocytic tumors, 10 WDLPS, 10 DDLPS	Tumor response: SD 10/10 (100%) WDLPS and 7/10 (70%) DDLPS	(60)
p53-MDM2 binding inhibitor SAR405838 (*Sanofi-Aventis)*	I	Solid tumours with no further effective standard treatment	>1	39 non-adipocytic tumors, 35 DDLPS	Tumor response: DDLPS 22/31 SD (71%)	(61)
p53-MDM2 binding inhibitor siremadlin and CDK4/6 inhibitor ribociclib *(Novartis)*	Ib	Locally advanced or metastatic WDLPS/DDLPS	>1	74	Tumor response: 3 PR, 38 SD	(59)
CDK4/6 inhibitor palbociclib *(Pfizer)*	II	Advanced WDLPS/DDLPS	>1	30 (plus 30 enrolled in expansion cohort)	Progression-free rate at 12 weeks: 57.2% Median progression-free survival: 17.9 weeks	(62)
multi-RTK inhibitor pazopanib (*Novartis*) and topoisomerase I inhibitor topotecan	II	Recurrent or metastatic, non-resectable STS, or metastatic or unresectable osteosarcoma	>1	106 non-adypocitic STS, 19 LPS, 28 osteosarcoma,	Overall response rate: 0%	(63)
multi-RTK inhibitor anlotinib (AL3818) (*Chia-tai Tianqing Pharmaceutical Co*)	II	Metastatic LPS	>1 (antacycline-based)	156 non adipocytic STS, 13 LPS	Progression-free rate at 12weeks: 63% Objective response rate: 7.7%	(64)
multi-RTK inhibitor pazopanib *(Novartis)*	II	Unresectable or metastatic LPS	>1	12 MLP, 27DDLPS, 2 pleomorphic LPS	Progression-free rate at 12weeks: 68%; Tumor response: 1 PR (2.4%), 17 SD (41.5%)	(65)
multi-RTK inhibitor regorafenib *(Bayer HealthCare)*	II	Metastatic LPS	>1	34 DDLPS, 12 myxoid/round cell LPS, 2 pleomorphic LPS	Overall response rate: 0% Progression-free survival: median, 2 months	(66)
XPO-1 inhibitor selinexor (*Karyopharm Therapeutics*)	II-III RCT	Advanced or unresectable DDLPS	2-5	285 (188 randomized to selinexor)	PFS: 2.8 vs 2.1 months (P=0.01) Tumor response: 5 PR (2.7%), 111 (59%)	(67)

MLPS, myxoid liposarcoma; PR, partial response; PFS, progression free survival; RCT, randomized controlled trial; SD, stable disease.

Additional preclinical studies suggested combination strategies including palbociclib and anti-IGF1R agents (e.g. R1507 or NVP-AEW541) which resulted in enhanced inhibition of cell cycle progression and metabolic activity (68). The combination of palbociclib with recombinant methionase heavily impacted tumor cell dependence on methionine in an orthotopic DDLPS PDX model resistant to doxorubicin (69).

Published findings of early clinical trials investigating MDM2 and CDK4 inhibitors in WD/DDLPS patients are summarized in **Table 2** (59–62).

## Potential targets and investigational therapies

### Receptor tyrosine kinases

Growth factor/RTKs drive and coordinate adipocytic differentiation by transducing stimulatory or inhibitory signals depending on multiple endogenous and environmental factors (70–72). The deregulated expression/function in LPS development and progression suggested some RTK axes as potential actionable targets. MET, AXL, KIT, and IGF1R were found overexpressed in WD/DDLPS cells versus normal adipocytes and pre-adipocytes (73) and genetic analyses evidenced common amplifications of *IGF2, IGF1R, ERBB3* together with *FGFR1, FGFR3* and *PDGFR* in WD/DDLPS (74–76). The more extensive amplification of genes encoding RTKs in DD than in WD components corroborated their role in promoting disease progression and the therapeutic potential of their targeting.

Overexpression and rare mutations of FGFRs, along with amplification of the adaptor protein FRS2 and the autocrine production of FGFs, contribute to the aberrant activation of the FGFR pathway in DDLPS (31, 74–82). Consistently, FGFR1 and/or FGFR4 overexpression has been associated with shorter disease-free survival and overall survival in WD/DDLPS patients (79). Treatment of DDPLS cells with the FGFR inhibitor erdafitinib (42, 43) (**Table 1**) reduced cell viability and induced apoptosis. Moreover, combination of erdafitinib with idasanutlin resulted in a synergistic antiproliferative effect, enhanced apoptosis and reduced DDLPS xenograft growth rate. In a case report, a patient with metastatic DDLPS refractory to multiple lines of conventional and targeted therapies experienced disease stabilization after erdafitinib treatment. These findings suggested FGFR1/FGFR4 expression as predictive biomarker in clinical trials investigating FGFR inhibitors.

The FGFR inhibitors infigratinib and LY2874455 (43, 44) exhibited *in vitro* and *in vivo* antitumor activity in a *FRS2*-amplified DDLPS experimental model originated from a high-grade metastatic tumor unresponsive to several conventional chemotherapeutics as well as to the MDM2 inhibitor RG7112 and palbociclib (**Table 1**) (81, 82). Differently from *FRS2* amplification, expression of FGFR signaling components (e.g. FGFs, FGFRL1) was suggested to modulate cell response to FGFR inhibitors. Moreover, activation of FGF/FGFR signaling is markedly affected by the co-accessory molecules heparan sulfate proteoglycans (HSPGs) (83, 84). In particular, the HSPG syndecan-1 was demonstrated to promote proliferation and inhibit differentiation of adipocyte progenitors (85, 86). Syndecan-1 was found overexpressed in DDPLS compared with normal adipose tissue and lipomas and its expression was controlled by FGF, a circuit that the FGFR inhibitor PD173074 could interrupt (86). FGF2 exerts a biphasic effect on adipogenesis with low concentrations enhancing adipogenesis (87). Moreover, the activity of adipokines such as FGF21 could be affected by the expression of the coreceptor Klotho which was found significantly reduced in DDLPS compared to healthy adipose tissue (88, 89). Notably, high levels of Klotho were associated with better survival in LPS patients (89). Klotho also modulates the insulin/IGF1 signaling (90), another positive regulator of adipocytic diffentiation (70, 71). Klotho overexpression reduced IGF1R signaling, decreased DDLPS cell proliferation and promoted gemcitabine-induced apoptosis (89). Analogously, the IGF1R inhibitor BMS-754807 increased gemcitabine-induced cell death confirming the implication of the RTK in DDLPS drug resistance. A combinatorial drug screening identified several synergistic target pairs in a DDLPS cell line including EGFR and IGF1R, IGF1R and CDK4, IGF1R and EGFR, IGF1R and STAT3. The combination of anti-IGF1R agents (e.g. R1507 or NVP-AEW541) with CDK4 inhibitors cooperatively suppressed the activation of proteins within the crucial AKT pathway (68).

The constitutive or HGF-induced Met activation enhanced DDLPS cell proliferation, migration and invasion. Accordingly, *MET* knockdown or pharmacological targeting by the Met inhibitors SU11274 and tepotinib (91) (**Table 1**) reduced DDPLS tumorigenic potential *in vitro* and *in vivo*. A high-throughput drug screening evidenced the antiproliferative activity of the Met inhibitors foretinib and crizotinib (45) (**Table 1**) on a panel of PDX-derived DDLPS cell lines (54). Interestingly, death receptor upregulation induced by Met inhibitor PHA-665752 pretreatment enhanced the antiproliferative and pro-apoptotic activity of TRAIL in DDLPS cell lines (92).

Inhibition of RTKs present on tumor and microenvironment cells was suggested to partecipate in the antitumor activity of multi-targeting RTK inhibitors (i.e. ponatinib, apatinib pazopanib) in DDPLS models (**Table 1**). Angiogenesis inhibition contributed to DDLPS PDX growth delay induced by pazopanib alone and in combination with doxorubicin (43, 45, 93–95). Clinical trials of anlotinib and pazopanib have been recently reported (**Table 2**) (63–65).

### Additional investigational therapies

WD/DDLPS are characterized by MDM2 overexpression associated with wild-type p53. MDM2-mediated ubiquitination downmodulates the tumor suppression function of p53 by promoting its nuclear export through exportin-1 (XPO1) and degradation (96). XPO1 overexpression, observed in LPS samples and cell lines (97), was recently confirmed in samples comprising the WD and DD components of primary tumors as well as the normal adipose tissue from DDLPS patients (98). These findings support targeting the nuclear export as a rational therapeutic approach for WD/DDLPS. The selective XPO1 inhibitor selinexor (46) (**Table 1**) decreased DDLPS cell growth by inducing cell cycle arrest and apoptosis (97, 98). Mechanistic studies showed that selinexor inhibited the IGF1R/AKT pathway activation by upregulating the expression of IGF binding protein 5 which acts as a tumor suppressor in DDLPS cells (97). Cell response to this drug was associated with a decrease of the survivin anti-apoptotic cytoplasmic pool (98). Selinexor significantly reduced the growth of a tumor xenograft from an established DDLPS cell line (97) and showed a moderate activity, anyway higher than doxorubicin, in three DDLPS PDXs displaying myogenic and rhabdomyoblastic heterologous differentiation (98). A phase III trials showed a small, though statistically significant, benefit for selinexor compared with placebo, suggesting also absence of the calcium and MDM2 binding protein CALB1 as a predictive biomarker for longer progression-free survival (67) (**Table 2**).

Recent reports highlighted the potential of the mitotic serine-threonine kinase Aurora A (AURKA) as therapeutic target. *AURKA* was found significantly upregulated in DDLPS compared to WDLPS and patients with high *AURKA* expression in tumors showed shorter recurrence-free survival (99). *AURKA* knockout and enzyme blockade by the inhibitors alisertib and AMG900 induced DDLPS cell cycle arrest and apoptosis (47) (**Table 1**). Alisertib also efficiently suppressed tumor xenograft growth (100, 101). Of note, DDLPS cell lines displayed heterogeneous sensitivity to AURKA/B inhibitors either alone or in combination with cytotoxic chemotherapeutics likely related to different tumor cell molecular characteristics (99–101).

## Discussion

Therapeutic options for WD/DDLPS are limited and patient outcomes remain unsatisfactory. The pathognomonic amplification of genes implicated in cell cycle and growth control has provided the rational bases for clinical evaluation of targeting agents. Despite high expectations, first reports recorded modest benefit from MDM2 and CDK4 inhibitors as single agents. MDM2 inhibitors evaluated in phase I/II trials showed disease stabilization in WDLPS and DDLPS (**Table 2**) (60, 61). The CDK4 inhibitor palbociclib resulted in favorable PFS and occasional tumor responses in a phase II trial for advanced WD/DDLPS (62). However, a real world experience in retroperitoneal diseases showed very limited clinical activity of single-agent palbociclib (102). Recently, trials are combining MDM2 and CDK4 inhibitors. A phase Ib study testing the combination of siremadlin and ribociclib recorded stable disease and manageable treatment-related toxicity supporting the feasibility of this approach (**Table 2**) (59).

Other recent trials have reported on pazopanib and other TKI inhibitors for liposarcomas (63–66) (**Table 2**). Extensive genomic analyses of WD/DDLPS and functional studies are currently actively exploring additional targets.

Among the recurrently amplified genes in WD/DDLPS, *HMGA2* deserves deeper investigation. In addition to its role as an oncoprotein associated with aberrant expression in several tumor types, HMGA2 promotes a cancer stem cell phenotype, chemoresistance, and is involved in adipogenesis at the clonal expansion step from preadipocytes to adipocytes (103–107). *HMGA2* transcript is overexpressed, or implicated in gene fusions, in DDLPS significantly more frequently than in their paired WDLPS samples (7). Efforts to identify HMGA2 inhibitors are ongoing (108, 109). Nonetheless, high levels of *MDM2* concurrent with low *HMGA2* amplification did correlate with low overall survival (110). The severe DDLPS rhabdomioblastic variant may harbor low HMGA2 amplification, which may have therapeutic implications as a lower expression of HMGA2 may result in higher drug sensitivity (98). In-depth investigation on the role of genetic alterations of *HMGA2*, and its relation with other players in DDLPS oncogenesis/progression, is needed to decipher its therapeutic relevance.

Recent insights into the WD/DDLPS molecular characteristics revealed an intrinsic heterogeneity reflecting early dedifferentiation and genomic instability. Although the mechanisms underlying dedifferentiation are not fully understood, additional genetic alterations, beyond already knew gene amplifications, may provide potential targets. Changes associated with progression from WDLPS to DDLPS include rearrangements of several chromosomal regions. Fusion transcripts appear more frequent in DDLPS than WDLPS (7) although few information exists about their association with histologic characteristics. Importantly, some of them are potentially druggable, such as fusions involving *NTRK* or *MAP2K6* genes described in case reports (111, 112).

Differentiation therapy may provide new therapeutic opportunities for DDLPS. Agonists of PPARγ, a key effector of adipogenesis, were shown to be effective inducers of re-differentiation in LPS preclinical models and clinical samples (113–115). Currently, while early clinical trials provided mixed results (116, 117), other trials of PPARγ ligands are underway in LPS patients. *PPARγ* is under the repressive control of the *FUS-CHOP* fusion oncogene in mixoid liposarcoma. Interestingly, the ability of trabectedin to displace *FUS-CHOP* from its targets promoters provided the rationale for the clinical evaluation of the PPARγ agonist pioglitazone (115). Although such differentiating approach appears of particular interest for mixoid liposarcoma, the maintenance of the adipocytic program could be pharmacologically exploited in at least subsets of DDLPS (73, 118).

Therapeutic progresses in LPS have been hampered by the disease rarity and the consequent challenge in designing prospective clinical trials and establishing predictive experimental models. In recent years, PDX models of WD/DDLPS, recapitulating tumor histology, biology and genetics, have been developed providing the opportunity to explore novel therapeutic approaches and relevant biomarkers (54, 98, 119). Patient-derived cell and xenograft models represent valuable tools to assess the therapeutic relevance of molecular alterations that, besides chromosomal amplifications shared by WDLPS and DDLPS, drive liposarcomagenesis and disease progression. Integrated “multi-omics” investigation will contribute to identify novel druggable vulnerabilities and synthetic lethal drug combinations eventually enhancing the development of innovative, biology-driven, effective treatments for WD/DDLPS patients.

## Author contributions

GC, SP and CL contributed to conception and design of the review. All authors contributed to the article and approved the submitted version.

## Funding

The authors acknowledge the support by “5x1000 Founds”, 2016 Italian Ministry of Health; Institutional grant BRI2017, GR-2019-12369175; FRRB-584-797,60; International Accelerator Award funded by AIRC [ID #24297]/Cancer Research UK [C56167/A29363]/Fundacion Científica, Asociacion Espanola Contra el Cancer [Foundation AECC-GEACC19007MA].

## Conflict of interest

The authors declare that the research was conducted in the absence of any commercial or financial relationships that could be construed as a potential conflict of interest.

## Publisher’s note

All claims expressed in this article are solely those of the authors and do not necessarily represent those of their affiliated organizations, or those of the publisher, the editors and the reviewers. Any product that may be evaluated in this article, or claim that may be made by its manufacturer, is not guaranteed or endorsed by the publisher.
